# New Remedies

**Published:** 1913-09

**Authors:** 


New Remedies. Acitrin is the trade name for ethyl phenylcinchoninate.

Acinophor is a mixture of cerium dioxide (3 parts) and thorium dioxid (1 part). It is used as an X-ray diagnostic.

Benzol has recently been advocated by Continental physicians in the treatment of leukæmia. The article referred to is *benzene,* $C_6H_6$, obtained by the fractional distillation of the light oil of tar, and not commercial benzol obtained from petroleum. Pure benzene is given in capsules containing ½ grain each benzene and olive oil, and has been found to reduce in a marked manner the number of leucocytes in the blood.

Brophenin is paraphenetidin bromisovalerylamino acetate, and occurs as a white tasteless powder. It is given as a febrifuge and as an analgesic in neuralgia, etc.

Ceolat is the cerium salt of a fatty acid, used as a solution, dusting powder, or ointment, in the treatment of wounds.

Cretaform is oxymethylcresol-tannin. It is an odorless powder used in the treatment of wounds.

Cuprase is a suspension of celloidal copper hydroxide, used for subcutaneous injection in cancer.

Lantol is a name given to colloidal rhodium (see Prescriber, Jan., p. 21). It is used for injection in cancer.

Mesbe is a preparation of *Sida rhombifolia,* recommended as a remedy for tuberculosis (*Münch. Med. Woch.,* Aug. 20, 1912; Jan. 21, 1913..

Ninhydrin is tri-keto-hydrin hydrate, and is used as a reagent for the detection of albumin, peptones, etc. (Meister, Lucius and Brüning).

Ortizon is a "solid hydrogen peroxide" prepared with urea, similar to *Hyperol* (see Prescriber, 1912, p. 59.)

Phylacogens are a series of products representing the secretions of specific micro-organisms. These secretions have been shown by Schafer to have an inhibitory influence on the growth of their own particular germ, just as acetic acid overcomes ordinary fermentation. Phylacogens are prepared for rheumatism, erysipelas, gonorrhoea, and mixed infection, by Parke, Davis & Co.

Scabosan is a nicotine salicylate soap, used for the treatment of scabies.

Thymacetol is the acetone ester of thymotic acid, recommended by Bachem (*Berlin. Klin. Woch.,* Oct. 28, 1912) as a local anaesthetic.

Vanadium Selenide, $Va_2Se_3$, is recommended by F. von Oefele as a remedy for cancer (*New York Med. Jour.,* Jan. 11, 1913).

—*Prescriber,* March, 1913.

The April issue of the *Prescriber* contains an elaborate discussion of Hormones; price, 27c. Address Edinburgh, Scotland.

